# Targeting of Acyl-CoA synthetase 5 decreases jejunal fatty acid activation with no effect on dietary long-chain fatty acid absorption

**DOI:** 10.1186/1476-511X-12-88

**Published:** 2013-06-14

**Authors:** Nahum Meller, Michelle E Morgan, Winifred PS Wong, Jessica B Altemus, Ephraim Sehayek

**Affiliations:** 1 Ephraim Sehayek, Genomic Medicine Institute, The Cleveland Clinic Foundation, 9500 Euclid Ave, Cleveland OH 44195, USA

**Keywords:** Small intestine, Fat absorption, Acsl5, Fatp4, LCFA, Acyl-CoA

## Abstract

**Background:**

The absorption of dietary long chain fatty acids (LCFA) largely occurs in the jejunum. LCFA are activated via conjugation with Coenzyme A (CoA), a reaction catalyzed by Acyl-CoA synthetases (ACS). Acyl-CoA sythesis is critical for dietary LCFA absorption; yet, the jejunal ACS enzymes that catalyze the reaction are largely unknown.

**Findings:**

High throughput mRNA sequencing of the mouse jejunum revealed that the expression of acyl-CoA synthetase 5 (Acsl5) and fatty-acid transport protein 4 (Fatp4) largely exceeded all other annotated ACS genes that activate LCFA. Interestingly, Acsl5 knockout (KO) mice displayed a decrease of 60% in jejunal total long chain acyl-CoA synthesis rate. Nevertheless, and despite of this decrease, dietary LCFA absorption and body-weight gain in response to high fat diet remained unaffected.

**Conclusion:**

Acsl5 is a major activator of dietary LCFA, yet in Acsl5 KO mice residual ACS activity is sufficient for maintaining a normal LCFA absorption. Our findings provide further evidence for a robust small intestine LCFA absorption capacity.

## Findings

### Background

Intestinal absorption of dietary long chain fatty acids (LCFA) is an important determinant of body weight and obesity-related metabolic disorders. LCFA absorption largely occurs in the jejunum where these nutrients undergo uptake across the brush border membrane of enterocytes. LCFA uptake is followed by a critical activation step through conjugation with coenzyme-A (CoA). In enterocytes, dietary LCFA-CoA conjugates are primarily utilized for triglycerides (TG) synthesis, packed into chylomicrons and secreted through the basolateral membrane [1,2]. LCFA conjugation with CoA is catalyzed by Acyl-CoA synthetases (ACS). Mammals possess 13 annotated ACS genes that activate LCFA and clustered in three different gene families: ACSL (acyl-CoA synthetase long chain), ACSBG (acyl-CoA synthetase bubblegum) and FATP (fatty acid transport proteins; also designated ACSVL or SLC27) [3-5]. The impact of each of these genes on intestinal absorption of LCFA is largely unknown.

## Methods

The methods used are described briefly at the figure legends. Detailed description of mice, diets and assays used in the study is presented in Additional files 1 and 2. All experiments involving mice were approved by the Cleveland Clinic Animal Care and Use Committee.

## Results

**Figure 1:**
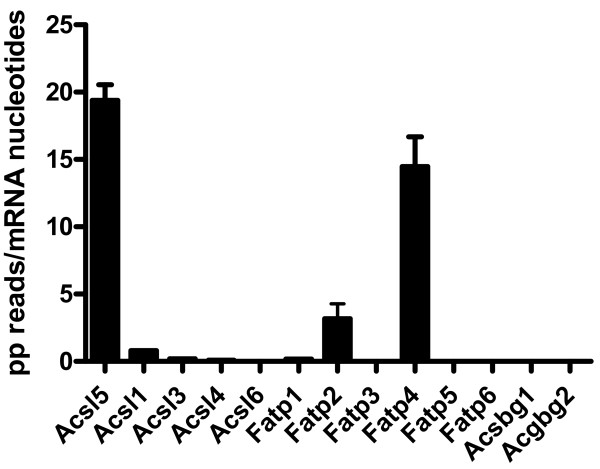
**Acsl5 and Fatp4 are the two predominant LCFA CoA**-**synthetases in mouse jejunum.** Five C57BL/6J females were fasted for 6 hours, sacrificed, the jejunum harvested, polyadenylated RNA isolated, cDNA synthesized and sequenced. Sequencing reads were aligned against a reference mouse genome. Gene expression levels were calculated as mean±SD of properly paired (PP)-end reads aligned to each gene after normalization for mRNA length in basepairs. Expression levels of the 13 annotated ACS genes is presented.

Expression of long chain acyl-CoA synthetase genes in C57BL/6J jejunum was determined by transcriptome shotgun sequencing analysis (mRNA-seq). As shown in Figure 1, out of the 13 annotated LCFA acyl-CoA synthetase genes, the number of properly paired reads that were aligned to Acsl5 and Fatp4 largely exceeded the number of reads that were aligned to the other genes. A previous study has shown that Fatp4 is dispensable for dietary fat absorption [6], therefore, we focused our efforts on elucidating the role of Acsl5. Quantitative PCR and Western blotting demonstrated that Acsl5 is preferentially expressed in the jejunum (Additional file 3). These results suggested a role for Acsl5 in determining the jejunal absorption of LCFA.

**Figure 2:**
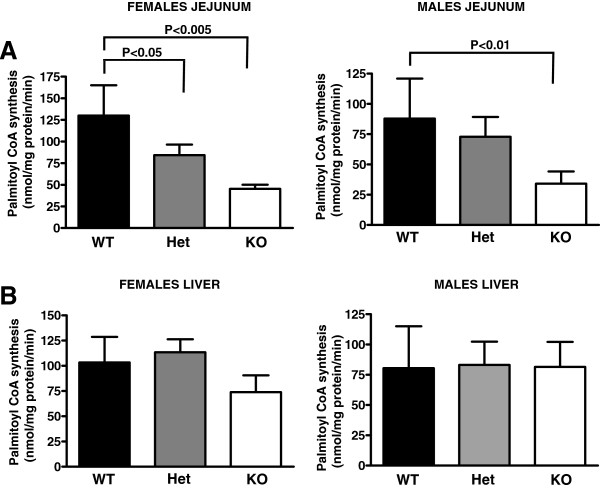
**Reduced total jejunal ACS activity in Acsl5 KO mice.** Jejunal mucosa (**A**) and liver tissues (**B**) from female or male Acsl5 wild type (WT), heterozygous (Het) or knockout (KO) mice, 5 animals per group, were harvested and homogenized. Equal amounts of protein were incubated in the presence of raiolabeled palmitic acid, ATP and CoA. Unconjugated fatty acid was extracted in heptane and aqueous phase radioactivity, containing the water soluble palmitoyl-CoA was determined by scintillation counting. Mean±SD palmitoyl-CoA synthesis rates are presented.

To determine the role of Acsl5 in jejunal LCFA-CoA synthesis and absorption, we have used mice that were targeted for this gene (characterization of targeted animals is found in Additional file 2). Jejunum mucosal homogenates were assayed for acyl-CoA synthesis rate by using palmitic acid, a prototype member of the long chain fatty acid family, as a substrate. As shown in Figure 2A, as compared to wild-type littermate controls, Acsl5-KO males and females displayed a decrease of 60% in jejunal palmitoyl-CoA synthesis rate. Animals heterozygous for the targeted allele displayed only a moderate 15-35% decrease in activity, which reached statistical significance only in females. When compared to the jejunum, liver Acsl5 expression is diminished (Additional file 3). Consistent with these findings, liver synthesis rates of palmitoyl-CoA in Acsl5 KO mice were fully conserved (Figure 2B).

**Figure 3:**
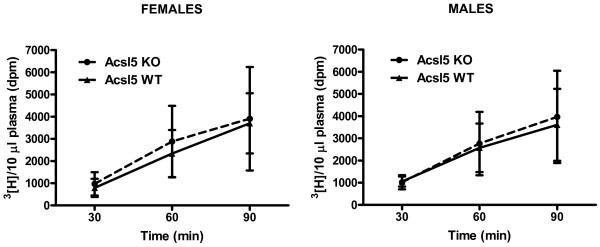
**Acsl5 KO mice display a normal dietary LCFA absorption from the intestine.** Acsl5 WT and KO mice, females or males, (5 per group), were fasted overnight and than intravenously injected with tyloxapol to inhibit vascular lipases. Thirty minutes later the mice received a gastric bolus olive oil supplemented with radiolabeled oleic acid and retroorbital sinus blood samples were collected at 30, 60 and 90 min post gavage. Plasma radioactivity was determined by scintillation counting.

To determine the effect of Acsl5 targeting on LCFA absorption, mice were fasted overnight, lipases activity inhibited through intravenous injection of tyloxapol, animals provided with a gastric bolus of olive oil supplemented with [^3^H]-radiolabeled oleic acid, and levels of the radioactive tracer were measured in plasma samples collected for the first 90 minutes post gastric gavage. As shown in Figure 3, as compared to littermate wild-type controls, Acsl5-KO males and females displayed undistinguishable plasma accumulation of the radiolabeled tracer. These finding suggested that targeting of the Acsl5 gene has no impact on intestinal LCFA absorption.

**Figure 4:**
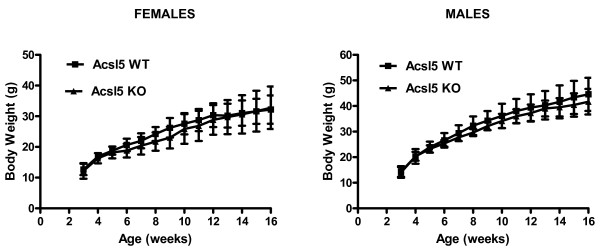
**Acsl5 KO display normal weight gain in response to high fat diet.** Acsl5 WT and KO mice, females or males, (5 per group), were fed a Western type diet for 13 weeks and body weights were recorded. No significant differences are observed between Acsl5 KO and WT animals.

To determine the effect of Acsl5 targeting on body-weight gain in response to high fat diet, mice were fed a Western-type diet for 13 weeks. As shown in Figure 4, Acsl5-KO males and females displayed weight gain that was indistinguishable of their littermate wild-type controls. Finally, targeting of Acsl5 had no effect on dietary cholesterol absorption, plasma cholesterol levels, plasma triglycerides or fasting plasma glucose levels (data not shown).

## Discussion

We identified Acsl5 as a major ACS gene expressed in the jejunum (Figure 1). Interestingly, while Acsl5 targeting resulted in a substantial decrease in jejunal total LCFA ACS activity (Figure 2) this decrease was not sufficient to reduce either LCFA absorption or body weight gain in response to high fat diet (Figures 3 and 4). As expression levels of other ACS genes in the jejunum remained stable (Additional file 2), these findings are consistent with a large capacity of the small intestine to absorb dietary LCFA. Therefore, even a reduction of 60% in total ACS activity is insufficient in affecting the absorption of LCFA even under conditions where the absorption process is stressed by the feeding of a high fat diet.

Our mRNA-Seq results suggest that jejunum Acsl5 and Fatp4 are expressed at a comparable level and together account for over 90% of the LCFA ACS transcripts in this tissue (Figure 1). A previous study reported, through targeting of Fatp4, that this enzyme is dispensable for intestinal fat absorption [6]. Unfortunately the authors have not reported the effect of Fatp4 targeting on jejunal LCFA-CoA synthesis rate. Interestingly, studies in newly born Fatp4-KO mice demonstrated ~10 fold inhibition in activation of the very long FA lignoceric acid (C24:0) but no inhibition of the LCFA palmitic or oleic acids [7]. These findings may suggest that Fatp4 is not a major determinant of LCFA absorption from the intestine. Nevertheless, studies in Acsl5/intestinal-Fatp4 double KO, that are beyond the scope of this report, are needed to clarify the combined effect of Acsl5 and Fatp4. It is of note that Fatp2, amongst the three highly-expressed LCFA ACS gene in the jejunum (Figure 1), may also contribute to LCFA absorption and the lack of phonotype in our Acsl5 KO mice. Finally, we cannot exclude the possibility that additional, not as yet identified, ACS genes may contribute to absence of phenotype in Acsl5 knockouts.

## Abbreviations

CoA: Coenzyme A; ACS: Acyl-CoA synthetase; LCFA: Long chain fatty acids; TG: Triglycerides; KO: Knockout; Acsl: Acyl-CoA synthetases long chain; Fatp: Fatty acid transport protein.

## Competing interests

The authors declare that they have no competing interests to report.

## Authors’ contributions

ES and NM designed the research and drafted the manuscript; MM, WW, JA and NM performed the experiments and analyzed the results. All authors read and approved the final manuscript.

## Supplementary Material

Additional file 1 **Methods.** Jejunum mRNA sequencing and analysis. Quantitative PCR. Western blotting Analysis. Total acyl-CoA synthesis assay. Dietary LCFA absorption assay. Statistical analysis. Click here for file

Additional file 2 **Acsl5 KO mice.** Animals and diets description. Acsl5 protein expression in WT, Heterozygote and KO mice. qPCR analysis of expression of major ACS genes and genes involved in fat absorption in Acsl5 KO and WT mice jejunum. Click here for file

Additional file 3 **Acsl5 is preferentially expressed in the jejunum.** RNA and protein levels of Acsl1 and Acsl5 in tissues with active fatty acid metabolism and along the longitudinal axis of the gastrointestinal tract. Click here for file

## References

[B1] Kindel T Lee DM Tso P The mechanism of the formation and secretion of chylomicrons Atheroscler Suppl 2010 11 11 16 20493784 10.1016/j.atherosclerosissup.2010.03.003

[B2] Iqbal J Hussain MM Intestinal lipid absorption Am J Physiol Endocrinol Metab 2009 296 E1183 E1194 10.1152/ajpendo.90899.2008 19158321 PMC2692399

[B3] Watkins PA Maiguel D Jia Z Pevsner J Evidence for 26 distinct acyl-coenzyme A synthetase genes in the human genome J Lipid Res 2007 48 2736 2750 10.1194/jlr.M700378-JLR200 17762044

[B4] Watkins PA Ellis JM Peroxisomal acyl-CoA synthetases Biochim Biophys Acta 1822 2012 1411 1420 10.1016/j.bbadis.2012.02.010 PMC3382043 22366061

[B5] Mashek DG Li LO Coleman RA Long-chain acyl-CoA synthetases and fatty acid channeling Future Lipidol 2007 2 465 476 10.2217/17460875.2.4.465 20354580 PMC2846691

[B6] Shim J Moulson CL Newberry EP Lin MH Xie Y Kennedy SM Miner JH Davidson NO Fatty acid transport protein 4 is dispensable for intestinal lipid absorption in mice J Lipid Res 2009 50 491 500 18843142 10.1194/jlr.M800400-JLR200 PMC2638106

[B7] Hall AM Wiczer BM Herrmann T Stremmel W Bernlohr DA Enzymatic properties of purified murine fatty acid transport protein 4 and analysis of acyl-CoA synthetase activities in tissues from FATP4 null mice J Biol Chem 2005 280 11948 11954 10.1074/jbc.M412629200 15653672

