# Supplementary material for: Targeting of Acyl-CoA synthetase 5 decreases jejunal fatty acid activation with no effect on dietary long-chain fatty acid absorption
Source: Lipids Health Dis. 2013 Jun 14;12:88. doi: 10.1186/1476-511X-12-88 (PMC3699395; doi:10.1186/1476-511X-12-88)
Supplement: Additional file 1 — Methods. Jejunum mRNA sequencing and analysis. Quantitative PCR. Western blotting Analysis. Total acyl-CoA synthesis assay. Dietary LCFA absorption assay. Statistical analysis. [file 1476-511X-12-88-S1.doc]

**Additional file 1:**

**Methods**

**Jejunum mRNA sequencing and analysis:** Five C57BL/6J females were fasted for 6 hours, anesthetized by intramuscular injection of ketamine/xylazine (174μg ketamine/5µg xylazine/g body weight) and sacrificed towards the end of the light phase of the light-dark cycle. The intestinal lumen was flushed with ice cold PBS and proximal 5cm of the jejunum (distal to the Treitz ligament) harvested and stored at -80°C. Jejunal total RNA was extracted by using the trizol reagent (Invitrogen, Carlsbad, CA) and polyadenylated RNA isolated by using the Oligotex mRNA kit (Qiagen, Valencia, CA). The polyadenylated mRNA was then processed according to Illumina protocol. Briefly, the mRNA was fragmented by using divalent cations and double stranded cDNA synthesized by using random primers. After conversion of overhangs into blunt ends, an adenosine base was added at the 3’ end and sequencing adapters ligated to the cDNA. Fragments of ~200bp were selected through gel excision and purified before amplification for 51bp paired ends sequencing by using the Illumina Genome Analyzer IIx. Sequencing reads were then aligned against a reference mouse genome (UCSC mm9) by using TopHat (v1.0.14; doi:10.1093/bioinformatics/btp120) and reads that are not properly paired were discarded from further analysis. For each RNA sample an average total of 30,391,087±1,736,964 properly paired reads were recovered. Jejunal acyl-CoA synthetase gene expression levels were calculated as mean±SD of properly paired-end reads aligned to each gene after normalization for mRNA length in basepairs.

**Quantitative PCR:** Tissues were harvested as described in the methods “Jejunum mRNA sequencing and analysis” section and kept at -80°C in RNA-later solution (Invitrogen) until processed. Tissues were homogenized using TissueLyser II (Qiagen) according to the manufacturer’s instructions. Total RNA extracted in TRIzol solution (Invitrogen), treated with DNAseI, and reverse transcribed by Superscript II (Invitrogen). Gene expression levels were determined by using SYBR Advantage qPCR Premix (Clonthech; Mountain View, CA) on a BioRad CFX384 platform. Specific gene expression was inferred from gene specific standard curves after normalization to polyadenylate-binding protein 1 (Pabpc1).

**2.4. Western blotting Analysis:** Tissues were harvested as described in the methods “Jejunum mRNA sequencing and analysis” section. Tissues were flash frozen in liquid nitrogen and polytron homogenized on ice in lysis buffer containing 50mM Tris pH 7.5, 150mM NaCl, 1% Triton X-100 and 1% protease inhibitor mix (Sigma P8340). Homogenates were centrifuged at 21,000 x *g* for 10min at 4^o^C, supernatants were collected and protein concentration was determined by using Pierce BCA assay (Cat# 23223). Clarified lysates were brought to equal protein concentration and supplemented with 5x SDS-PAGE sample buffer (final concentration 62.5mM Tris, 6.8, 2% SDS, 10% glycerol, 5% β-mercaptoethanol). Proteins were resolved by SDS-PAGE, transferred onto PVDF membranes, blocked in PBST 5% non-fat dry milk and probed with antibodies for Acsl5 (Santa Cruz biotechnology; N17 #48002), Erk1/2 (Cell Signaling; #9102), Acsl1 (Cell signaling; #4047S), and Gapdh (Cell signaling; #2118S). The intensity of protein signals was quantified by densitometry by using the ImageJ software.

**Total acyl-CoA synthesis assay.** Liver and Jejunum tissues were harvested as described above, the jejunum was wide opened longitudinally and the mucosa was gently scraped on ice. Tissues were homogenized by 10 strokes in Dounce homogenizer in a buffer containing 250mM sucrose, 10mM Tris-HCl, pH7.4, 1mM EDTA, 1mM DTT and 1% protease inhibitor mix (Sigma P8340). The lysate was spun 5 min at 455 x *g* and the supernatant collected and flash frozen on liquid nitrogen. Equal amounts of protein were added to a pre-warmed acyl-CoA activity buffer (175mM Tris-HCl, 7.4, 5mM DTT, 0.01mM EDTA, 8mM MgCl_2_, 0.5mM Triton X-100, 450µM palmitic acid (Sigma P5585), 50µM 1-[^14^C] palmitic acid (60mCi/mmol; Perkin Elmer, Waltham, MA), 10mM ATP and 250uM CoA (Sigma C4282)), samples incubated for 5 minutes at 37°C and reactions stopped by adding an equal volume of Dole’s reagent (isopropanol: heptane: 1M MgSO_4_ 80:20:2 v/v). Samples were vortexed, 0.5 volume of ddH_2_O was added, and unconjugated fatty acids extracted 3 times in heptane. Aqueous phase radioactivity, containing the water soluble acyl-CoAs was determined scintillation counting.

**Dietary LCFA absorption assay:** Mice were fasted overnight, and lipases activity was inhibited through intravenously injected of 2.2µl/g body weight of 20% tyloxapol (Sigma T8761) in PBS solution. Thirty minutes later the mice received a gastric bolus of 50µl olive oil supplemented with 5µCi of 9,10-^3^H(N) [^3^H]-labeled oleic acid (45.5mCi/mmol; Perkin Elmer) and retroorbital sinus blood samples were collected at 30, 60 and 90 min post gavage. Plasma samples (10μl) were subjected to scintillation counting.

**Statistical analysis:** Group differences in gene expression, total acyl-CoA synthetase activity, dietary LCFA absorption, and body weight were analyzed by using student’s unpaired t-test. Unless otherwise indicated, values throughout this manuscript are expressed as mean±SD.
