# Supplementary material for: Targeting of Acyl-CoA synthetase 5 decreases jejunal fatty acid activation with no effect on dietary long-chain fatty acid absorption
Source: Lipids Health Dis. 2013 Jun 14;12:88. doi: 10.1186/1476-511X-12-88 (PMC3699395; doi:10.1186/1476-511X-12-88)
Supplement: Additional file 2 — Acsl5 KO mice. Animals and diets description. Acsl5 protein expression in WT, Heterozygote and KO mice. qPCR analysis of expression of major ACS genes and genes involved in fat absorption in Acsl5 KO and WT mice jejunum. [file 1476-511X-12-88-S2.pdf]

Additional File 2.

Wild type and Acsl5 KO mice

Animals and diets: C57BL/6J mice were purchased from Jackson Laboratories (stock # 000664). Mice were housed in a temperature-controlled room with a 14/10 hours light/dark cycle at the Cleveland Clinic Biological Resources Unit. Unless otherwise indicated, mice were fed a standard chow diet (18% protein, 44% carbohydrates, 6% fat; Harlan Laboratories, Teklad cat # 2918) and subjected to experiments at 12-14 weeks of age. In specific experiments mice were fed a Western type diet (17% protein, 49% carbohydrates, 21% fat; Harlan Laboratories, Teklad cat # 88137). Mice targeted for the Acsl5 gene on a mixed C57BL/6X129S genetic background were purchased from MMRRC (strain: C57BL/6;129S5-Acsl5tm1Lex/Mmucd). This strain has a deletion of exons 15-17 resulting in truncation of the catalytic site. Heterozygous Acsl5-KO mice were intercrossed to produce littermate wild type, heterozygous, and homozygous mice that were used for experiments. These experiments were approved by the Cleveland Clinic Animal Care and Use Committee

Acsl5 mice are characterized by complete absence of jejunal Acsl5 protein whereas heterozygous animals display an intermediate 40% reduction in the protein level of this enzyme (Additional Figure 1A). Acsl5-KO were fertile, displayed a Mendelian segregation of the targeted allele, a normal jejunal histology (not shown), and no changes in jejunal expression of genes involved in acyl-CoA synthesis (including Acsl1, Acsl3, Fatp2 and Fatp4) or in fat absorption (including Dgat1, Mogat2, Mttp, apoB or Hnf4α; Additional Figure 1B and 1C).

Additional Figure 1.

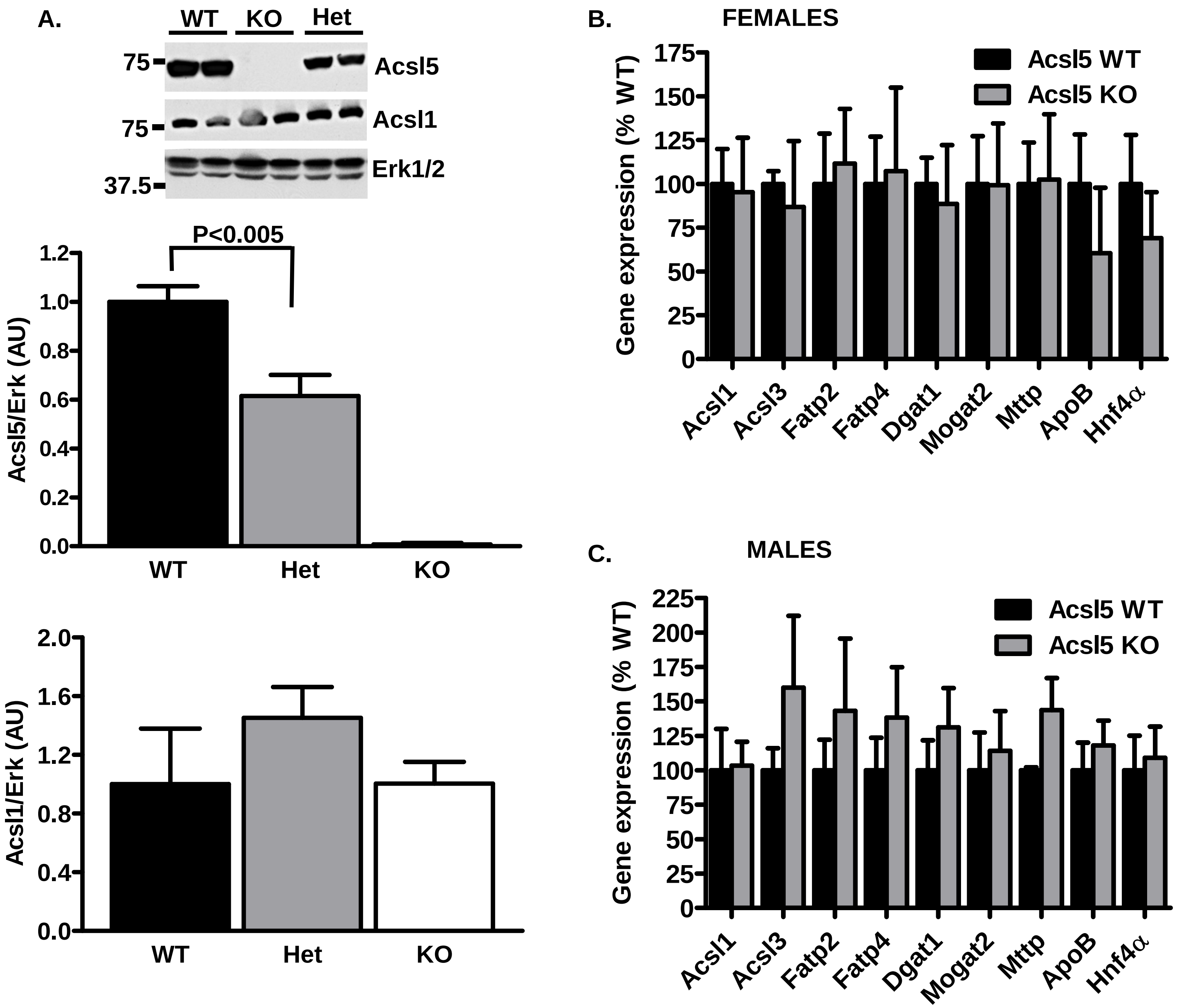

Legend Additional Figure 1. **A.** Acsl5 and Acsl1 expression levels in the jejuna of wild type (WT), Acsl5 knockout (KO), and Acsl5 heterozygous (Het) mice. Jejunum tissue was harvested and processed for Acsl5 and Acsl1 Western blotting and densitometry analysis as detailed in Additional file 1. N=5 female mice per group. Shown are samples of 2 animals per group. **B, C.** Jejunal expression of genes in wild-type (Acsl5 WT) and Acsl5-KO mice. The jejunum tissue was harvested, RNA extracted, and gene expression determined as detailed in Additional file 1. N=5 mice per group. Dgat, diacylglycerol acyltransferase; Mogat, monoacylglycerol acyltransferase; MTTP, Microsomal triglyceride transfer protein; apoB, Apolipoprotein B; Hnf4α, hepatocyte nuclear factor 4 alpha.
