# Supplementary material for: Targeting of Acyl-CoA synthetase 5 decreases jejunal fatty acid activation with no effect on dietary long-chain fatty acid absorption
Source: Lipids Health Dis. 2013 Jun 14;12:88. doi: 10.1186/1476-511X-12-88 (PMC3699395; doi:10.1186/1476-511X-12-88)
Supplement: Additional file 3 — Acsl5 is preferentially expressed in the jejunum. RNA and protein levels of Acsl1 and Acsl5 in tissues with active fatty acid metabolism and along the longitudinal axis of the gastrointestinal tract. [file 1476-511X-12-88-S3.pdf]

Additional file 3

Acsl5 is preferentially expressed in the jejunum

Amongst members of the Acsl gene family, Acsl1 and Acsl5 presented the highest jejunal expression levels. Therefore, we examined the expression of these genes in tissues with active fatty acid metabolism. As shown in Additional Figure 2A, the expression of Acsl1 mRNA in the heart, liver and brown adipose tissues (BAT) exceeded by 20-40 folds the levels displayed in the jejunum. In contrast, the expression level of Acsl5 in the jejunum exceeded by 3-29 folds the levels in the other tissues. Interestingly, the differences at the protein level were even more striking. Therefore, whereas Acsl5 protein was clearly detected in Western blotting of the jejunum homogenates, the signal of this enzyme in the other tissues was very weak. Acsl1 expression displayed a reversed pattern with a clear signal detected in all tissues but a very weak signal in the jejunum (Additional Figure 2B). We also examined the expression of Acsl1 and Acsl5 across the longitudinal axis of the gastrointestinal tract. As shown in Additional Figure 2C, whereas Acsl1 expression was detected throughout the gastrointestinal tract, the expression of Acsl5 was restricted to the jejunum, with no evidence for expression of this enzyme in the ileum or the colon.

Additional figure 2.

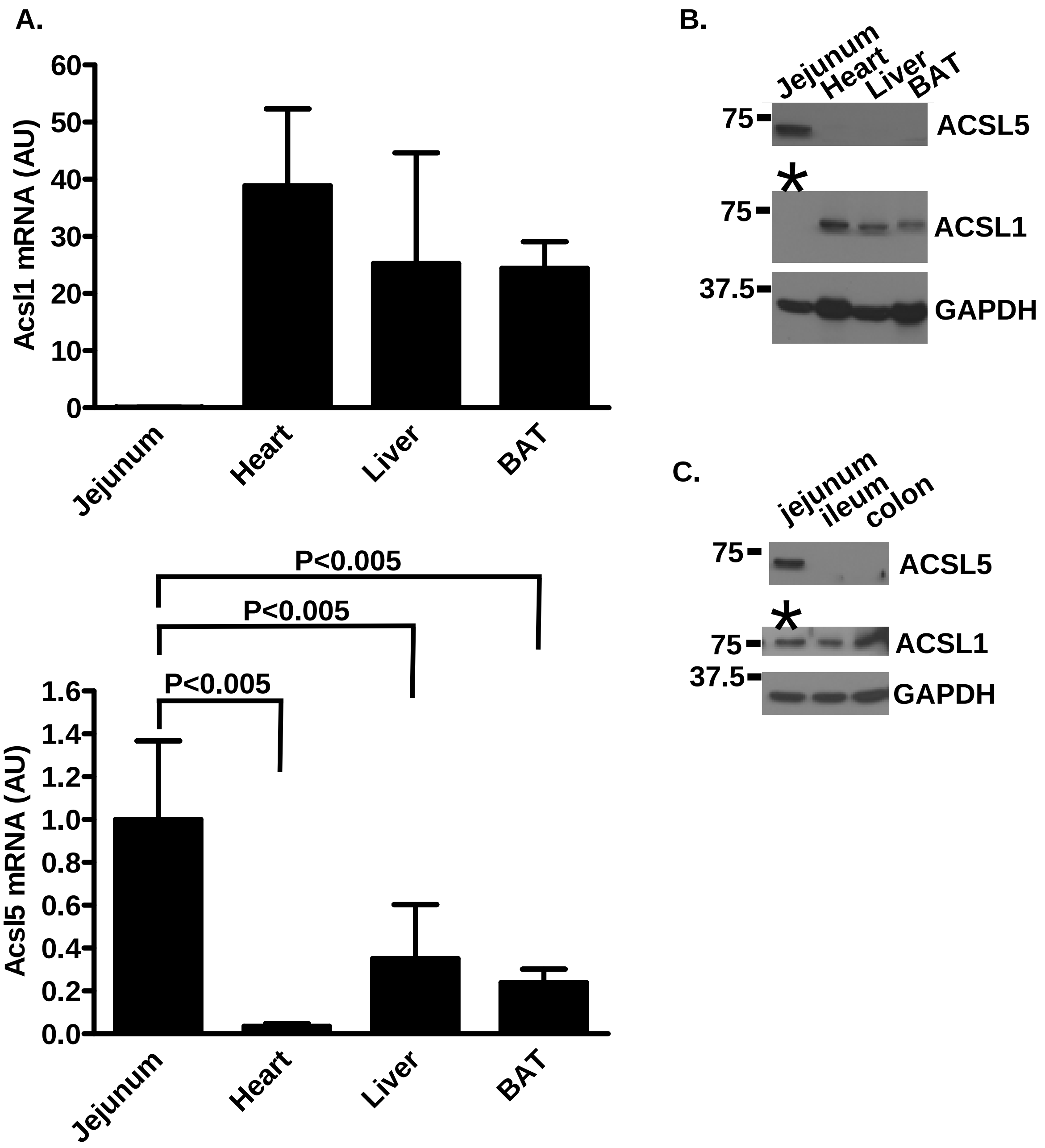

Legend Additional Figure 2. RNA and protein levels of Acsl1 and Acsl5 in tissues with active fatty acid metabolism (A and B) and along the longitudinal axis of the gastrointestinal tract (C). Tissues RNA and proteins were extracted and processed for qPCR and Western blotting as described in Additional file 1. BAT= brown adipose tissue. N=5 (qPCR) or N=3 (protein) male mice per group. * Discordance in jejunum Acsl1 expression level in Figures 2B and 2C is due to a longer blot exposure time in Figure 2C.

Discussion

Mashek et al. examined the expression of different Acsl isoforms in the rat and reported a predominant expression of Acsl5 mRNA in the liver, BAT and the duodenum [1]. The results in Additional Figure 2A support these findings, however, analysis at the protein level clearly indicates that Acsl5 expression level in the jejnum largely exceeds the expression in the liver and the BAT tissues (Additional Figure 2B). Indeed, under Western blotting conditions where the Acsl5 protein signal in the jejunum homogenates is unsaturated, very weak signals is detected in the liver and BAT homogenates (Additional Figure 2B). These findings are in agreement with previous studies in humans that reported ACSL5 as an abundant protein of the small intestine mucosa [2].

References

1. Mashek DG, Li LO, Coleman RA: **Rat long-chain acyl-CoA synthetase mRNA, protein, and activity vary in tissue distribution and in response to diet.** *J Lipid Res* 2006, **47:**2004-2010.
2. Gassler N, Kopitz J, Tehrani A, Ottenwalder B, Schnolzer M, Kartenbeck J, Lyer S, Autschbach F, Poustka A, Otto HF, Mollenhauer J: **Expression of acyl-CoA synthetase 5 reflects the state of villus architecture in human small intestine.** *J Pathol* 2004, **202:**188-196.
